# COVID-19 detection from chest X-ray images using CLAHE-YCrCb, LBP, and machine learning algorithms

**DOI:** 10.1186/s12859-023-05427-5

**Published:** 2024-01-17

**Authors:** Rukundo Prince, Zhendong Niu, Zahid Younas Khan, Masabo Emmanuel, Niyishaka Patrick

**Affiliations:** 1 Department of Computer Science and Technology, Beijing Institute of Technology, Beijing, China; 2 Computer Science and Information Technology, University of Azad Jammu and Kashmir, Kashmir, Pakistan; 3 Software Engineering, African Center of Excellence in Data Science(ACE-DS), and the African Center of Excellence in Internet of Things(ACEIoT), University of Rwanda, Kigali, Rwanda; 4 Computer and Information Sciences, University of Hyderabad, Hyderabad, India

**Keywords:** COVID-19, YCrCb, CLAHE, HE, Max–Min filter, LBP

## Abstract

**Background:**

COVID-19 is a disease that caused a contagious respiratory ailment that killed and infected hundreds of millions. It is necessary to develop a computer-based tool that is fast, precise, and inexpensive to detect COVID-19 efficiently. Recent studies revealed that machine learning and deep learning models accurately detect COVID-19 using chest X-ray (CXR) images. However, they exhibit notable limitations, such as a large amount of data to train, larger feature vector sizes, enormous trainable parameters, expensive computational resources (GPUs), and longer run-time.

**Results:**

In this study, we proposed a new approach to address some of the above-mentioned limitations. The proposed model involves the following steps: First, we use contrast limited adaptive histogram equalization (CLAHE) to enhance the contrast of CXR images. The resulting images are converted from CLAHE to YCrCb color space. We estimate reflectance from chrominance using the Illumination–Reflectance model. Finally, we use a normalized local binary patterns histogram generated from reflectance (Cr) and YCb as the classification feature vector. Decision tree, Naive Bayes, support vector machine, K-nearest neighbor, and logistic regression were used as the classification algorithms. The performance evaluation on the test set indicates that the proposed approach is superior, with accuracy rates of 99.01%, 100%, and 98.46% across three different datasets, respectively. Naive Bayes, a probabilistic machine learning algorithm, emerged as the most resilient.

**Conclusion:**

Our proposed method uses fewer handcrafted features, affordable computational resources, and less runtime than existing state-of-the-art approaches. Emerging nations where radiologists are in short supply can adopt this prototype. We made both coding materials and datasets accessible to the general public for further improvement. Check the manuscript’s availability of the data and materials under the declaration section for access.

## Background

In 2019, the globe witnessed one of the most widespread outbreaks, the coronavirus (COVID-19). In January 2020, the World Health Organization (WHO) conceded COVID-19 as a public health emergency of international concern. WHO reported about 482.34 million infected cases with 6.15 million fatalities worldwide around March 2022 [1]. COVID-19 comes from SARS-CoV-2, one of the $\beta$-coronavirus family.It is one of the most transmissible, contagious, and infectious viruses among those implicated in Middle East Respiratory Syndrome (MERS) and Severe Acute Respiratory Syndrome (SARS). Most infections occur by respiratory droplets, touching (nose, mouth, and eyes), or any other form of close contact [2].

Timely detection and diagnosis of the virus increase the prognostic probability of preventing its transmission. Thus, fewer infected cases and fatalities may occur. Nowadays, healthcare systems use reverse transcription-polymerase chain reaction (RT-PCR) [2]. RT-PCR yielded accurate results, associated with some limitations. Kameswari et al. [3] reported a limited sensitivity during the early stages of the disease. Purohit et al. [4] proved that the RT-PCR approach provides false-positive rates higher than expected. Another drawback of this method, it only recognizes viral RNA presence with the anticipation that a patient who recovered from COVID-19 may be detected as an infected one [5, 6].

To overcome RT-PCR limitations, researchers suggested deep learning, machine learning, and transfer learning models [7–9]. Nevertheless, deep learning and machine learning models [10, 11] exhibit notable limitations, such as the need for large datasets to train, expensive computational resources graphical processing unit (GPUs), more extensive trainable parameters, feature vector size, longer running, training, and testing time. Conversely, transfer learning models yielded negative transfer and overfitting concerns [12].

From the background of this work, it is within the scope to address the above limitations. We proposed a simple, coherent, and computationally efficient model to address some earlier drawbacks. The suggested method requires a smaller feature vector size, operates on a commodity CPU system, and exhibits less running time to detect COVID-19 chest X-ray (CXR) images and pneumonia diseases.

### Our major contribution

- In literature, high-performance deep learning methods are complex and require extensive data to train/test and run on expensive GPUs. However, we proposed a simple novel approach using CLAHE, YCrCb, and reflectance features, which runs on commodity hardware, and a low-cost CPU (see Table 7).
- The proposed model demonstrates experimentally that CLAHE, YCrCb, and reflectance features improve previously studied handcrafted features. It uses basic handcrafted features and shows comparable performance results to state-of-the-art methods based on complex deep learning models (see Table 8).
- Finally our model has a significant advantage in making real-time clinical decisions. It achieved high classification accuracy, detecting COVID-19 and Pneumonia, using less run time, and a smaller feature vector size of 25 (see Table 2).

The manuscript is structured in the following manner. Related works are given in “Related works” section. “Methods” section showcases the methods used. “Experimental results” and “Results” sections explain in detail the experimental results. Finally, comparative results and the conclusion are discussed in “Comparative results” and “Conclusion” sections.

## Related works

Subramanian et al. [13] showed that transfer learning outperforms all proposed models. InceptionV3, DenseNet201, and Mobile-NetV2 attained better accuracy, while SqueezeNet and VGG19 reported high specificity. The study conducted by Purohit et al. [4] suggested a deep learning model using a multi-image augmentation technique. This work reported an accuracy of 98.97%. To facilitate COVID-19 detection, Bhattacharyya et al. [14] proposed a deep learning-based generative model (C-GAN). This reduces the complexity of images when computing discriminatory features. An accuracy of 96% was reported. The work proposed by Ismael et al. [15] adopted a deep feature extraction pre-trained on deep CNN models. They used SVM with a linear kernel function as their classifier. An accuracy score of 94.7% was obtained. A deep learning model to detect COVID-19 from CXR, CT scans, and ultrasound images was introduced by Horry et al. [16]. VGG19 model detected COVID-19, pneumonia, and normal images with an accuracy of 86%, 84%, and 100%, respectively. Alshayeji et al. [17] proposed a computer-aided diagnosis (CAD) technique to classify COVID-19 and normal lungs. Their method performed a three-class semantic segmentation of the lung CT image in infected regions. They presented a global accuracy of 99.47%, a mean accuracy of 94.04%, and a mean IoU (intersection over union) of 0.8968.

Shah et al. [18] proposed a hybrid deep learning method, combining a convolutional neural network (CNN) and gated recurrent unit (GRU) for detecting viral diseases in chest X-rays (CXRs).CNN extracted features and the GRU acted as a classifier. After training on 424 CXR images with 3 classes, the model achieved a precision, recall, and f1-score results of 0.96, 0.96, and 0.95, respectively. The work conducted by Bhyuyan et al. [19] proposed a full-resolution convolutional network (FrCN) to detect COVID-19 from CT scan images. Applying a fourfold cross-validation test, FrCN, with an accuracy of 99.9%, performed better than other state-of-the-art models. Khan et al. [20] proposed a novel SB-STM-BRNet CNN model, which incorporated squeezed and boosted (SB) and dilated convolutional-based split-transform-merge (STM) block to detect COVID-19. This model reported an accuracy of 98.21%. Khalifa et al. [11] developed a deep-learning semantic segmentation model for COVID-19 detection based on the encoder and decoder concepts. Their experimental results reported a global accuracy of 99.3% and a Weighted Intersection over Union (WIoU) of 98.7%. Khan et al. [21] proposed two novel deep learning frameworks: deep hybrid learning (DHL) and deep boosted hybrid learning (DBHL), using machine learning (ML) classifiers to detect COVID-19. The DBHL framework, merging the two-deep CNN features, reported an accuracy of 98.53%.

Mubarak et al. [9] proposed an integration of VGG-19 and a handcrafted LBP model to train KNN and SVM classifiers. An overall accuracy of 99.4% was reported. Adimoolam et al. [10] proposed a model which predicts and classifies diseases using chest X-ray images. Their model extracts textual and morphological features. Aggarwal et al. [2] proposed to use MobileNetV2, ResNet50, InceptionV3, NASNetMobile, VGG16, Xception, InceptionResNetV2, and DenseNet121. These transfer learning models were fine-tuned by adding a new set of layers to increase their performance. DensNet121 achieved an accuracy of 97% on the first dataset. Whereas, MobilNetV2 generated an overall accuracy of 81% on the second dataset. Zammit et al. [7] developed a generative model (shared variational auto-encoder) using a five-layer deep hierarchy of latent variables and deep convolutional mappings.

Machine learning models have emerged as powerful tools to diagnose and detect COVID-19 disease. The work by Kwekha-Rashid et al. [22] introduced a review study highlighting the importance of machine-learning algorithms in detecting COVID-19. Barstugan et al. [8] developed a classification model to detect COVID-19 from CT images using handcrafted features. They applied grey level co-occurrence, zone matrix (GLCM/GLZM), and local directional pattern (LDP) as feature extraction techniques. The work conducted by Kassania et al. [23] compared popular deep learning models. DensNet121 feature extractor with Bagging Tree classifier reported an overall accuracy of 99%. Kumar et al. [24] presented a machine-learning model based on deep features extracted using ResNet152. This approach achieved an accuracy of 97.3% using Random Forest and 97.7% using XGBoost. Mydukuri et al. [25] proposed a model based on LSRGNFM-LDC (least-square regressive Gaussian neuro-fuzzy multi-layered data classification) method. Their method uses a Deming least square regressive to extract features.

It is apparent from the literature review that most of the above models achieved good performances. Nevertheless, they used numerous integrated algorithms, larger feature vectors size, and many trainable parameters [2, 11, 16]. As a result, it engenders more running time and expensive computational resources (GPUs).

## Methods

**Fig. 1:**
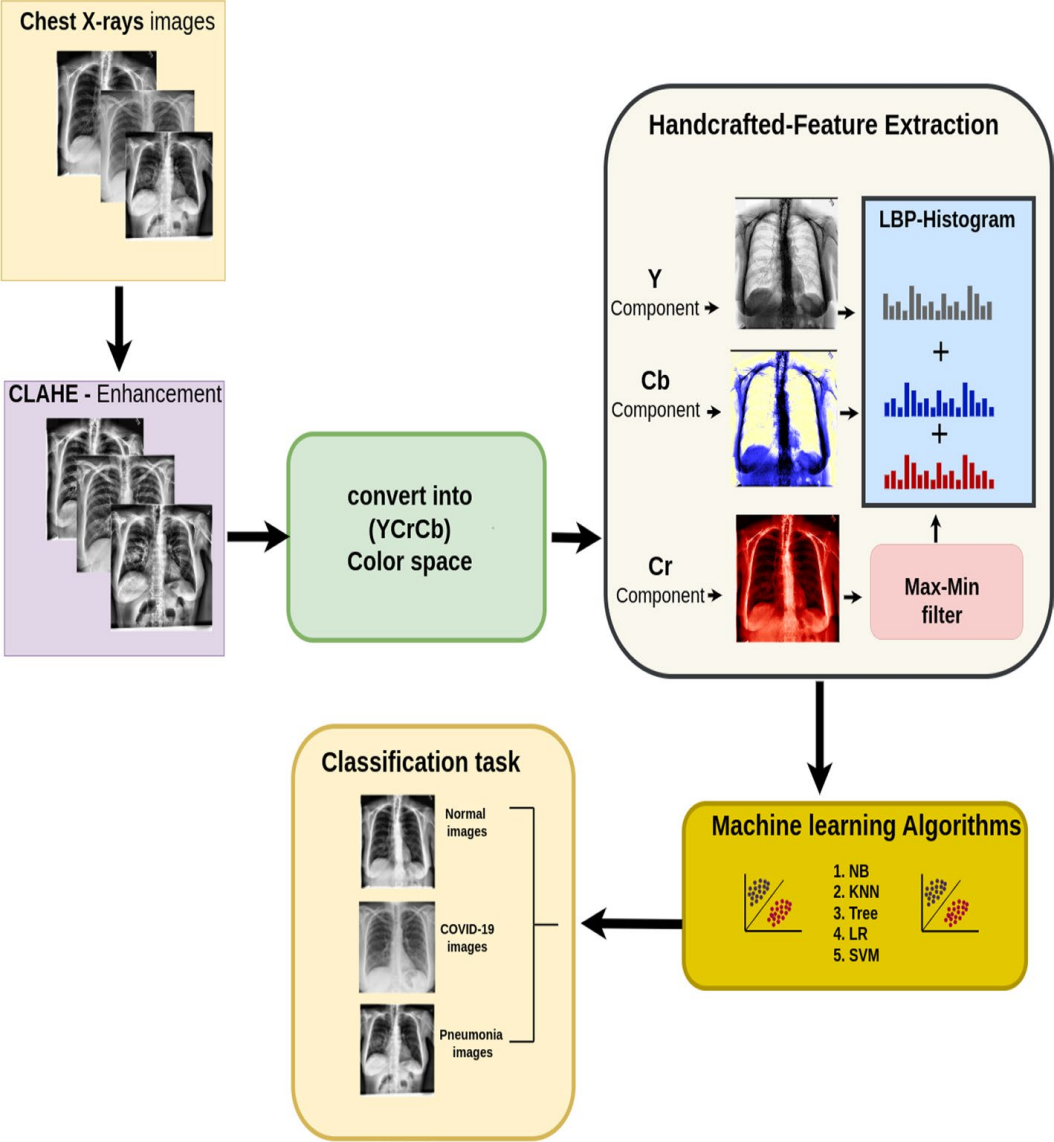
Flowchart of the proposed model

We propose a simple, coherent, and efficient computational technique to address the above drawbacks. We adopted CLAHE-YCrCb image processing technique [26, 27], the Illumination–Reflectance model [28], LBP [29], and machine learning classifiers [30]. First, Contrast Limited Adaptive Histogram Equalization is applied to enhance the contrast of the chest X-ray images. Second, we convert the output images from CLAHE into YCrCb color space. Third, we estimate reflectance from chrominance using the Illumination–Reflectance model. Finally, a normalized local binary patterns (LBP) histogram, generated from reflectance (Cr) and YCb, is used as the classification feature vector. DT, NB, SVM, KNN, and LR Machine learning algorithms are used to classify COVID-19, normal, and pneumonia CXR images. The overview of the proposed method is highlighted in Fig. 1.

### Contrast limited adaptive histogram equalization (CLAHE)

CLAHE improves the appearance of an image and increases the performance of subsequent tasks, such as image segmentation, analysis, and object detection. Enhancing an image strengthens its quality and provides a better computational analysis. CLAHE performs better in image deblurring, noise removal, and contrast enhancement. It expands the gray level’s dynamic range [26, 31, 32]. This study adopted CLAHE to implement a local contrast image enhancement, enhancing image quality and enabling a better computational detection of COVID-19 from CXR images.

**Fig. 2:**
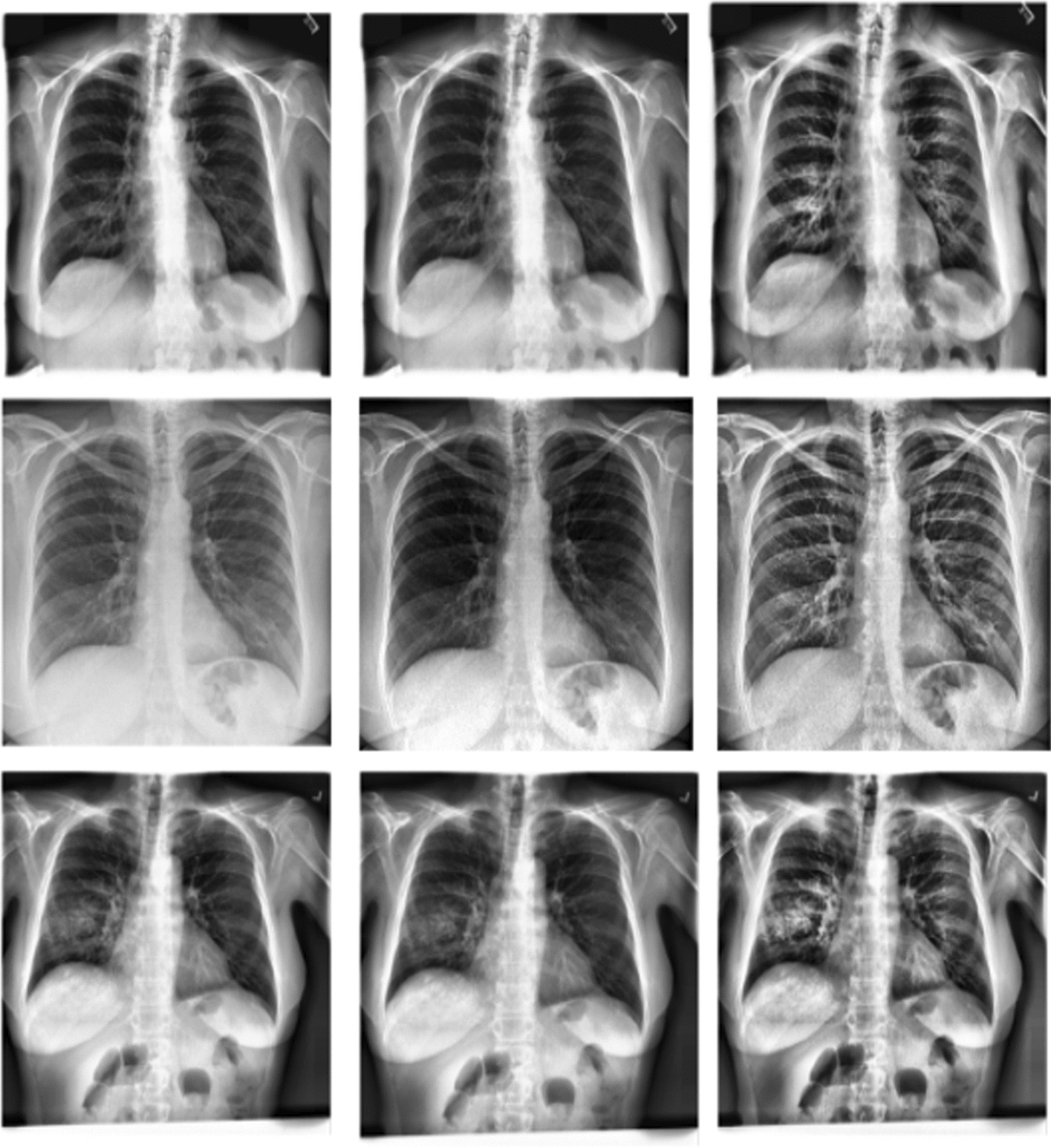
*Rowwise* First row: Normal images. Second row: COVID-19 images. Third row: pneumonia images. *Columnwise* First column: not enhanced images. Second column: images enhanced with HE. Third column: images enhanced with CLAHE

Figure 2 depicts the histogram equalizer (HE) and CLAHE enhancement on Normal, COVID-19, and pneumonia CXR images.

### Converting CLAHE’s CXR images into YCrCb

*YCrCb* possesses a luminance *Y* and a chrominance *CrCb*. *CrCb* is red-difference and blue-difference from the chrominance constituents. Luminance, on the other hand, is an intensity constituent [28]. Equation 1 highlights the luminance. Equations 2 and 3 depict the chrominance Cb and Cr.

1 $$\begin{aligned} Y&= 0.299 * R + 0.587 * G + 0.114 * B \end{aligned}$$

2 $$\begin{aligned} Cb&= 0.492(B - Y) \end{aligned}$$

3 $$\begin{aligned} Cr&= 0.877(R - Y) \end{aligned}$$

Several studies showed that chrominance is a suited component for detecting objects in medical images. In [33], authors identified skin color using three-color spaces, namely: *HSV*, *YCrCb*, and normalized *RGB*. This study detected the skin pixel with an accuracy of 91%. Using chrominance color space techniques to detect objects outperforms the existing model in facial recognition [34]. The Chroma component is an adequate feature for edge detection and localizing objects [34]. We opted to use CLAHE and chroma based on the advantages discussed above.

### Extracting reflectance component from chroma

The Illumination–Reflectance model highlights how objects interact with light [33]. It is used in image enhancement applications that rely on the Homomorphic filter [35] or retinex [36]. This model presumes that each pixel intensity shows the quantity of light reflected by a specific object. This corresponds to the product of illumination and the scene reflectance component of an object.

*L*, *R*, and *F* depict the illumination, reflectance, and image formation, respectively [37]. Niyishaka et al. [28] highlighted that *L* relates to the low-frequency component, and *R* relates to the high-frequency component. Moving into the log domain (ln), to separate the illumination and reflectance components, we can turn a multiplicative component into an additive one. The following equation highlights the details. Equation 4 shows multiplicative component. Equation 5 shows the process of moving into the log domain (ln). Equation 6 turns a multiplicative component into an additive one.

4 $$\begin{aligned} F(x,y)&= L(x,y)R(x,y) \end{aligned}$$

5 $$\begin{aligned} \ln (F(x,y))&= \ln {(L(x,y)R(x,y))} \end{aligned}$$

6 $$\begin{aligned} \ln (F(x,y))&= \ln {((L(x,y))}+\ln {(R(x,y))} \end{aligned}$$

### Max–Min filter application

Fasihi et al. [38] demonstrated that the sharp edges of the image are located in high-frequency bands. Perceiving that *R* relates to the high-frequency component [28], we use the Max–Min filter that blurs CXR images by keeping essential edges.

From this perspective, the Max–Min filter (approximation of an edge-preserving filter) was applied to extract *Cr* from *YCrCb*. The final image is the estimated reflectance constituent *R*. Because both *Cr* and *Cb* are Chroma components, their performances have been compared; *Cr* performed better than *Cb*. Equation 7 define the Max–Min filter. F(x, y), represents the pixel value at coordinates (x, y) in the output (filtered) image. Sxy {K(i, j)}: This expression involves a neighborhood Sxy centered at the pixel (x, y) in the original image. K(i, j), represents the pixel value at coordinates (i, j) in the original image, within the defined neighborhood Sxy.

7 $$\begin{aligned} f(x,y)= max_{(i,j)\in S_{xy}} \{ K(i,j) \}, \;\;\;\;f(x,y)= min_{(i,j)\in S_{xy}} \{ K(i,j) \} \end{aligned}$$

Algorithm 1 uses *Cr* with the Max–Min filter to estimate reflectance component *R*.
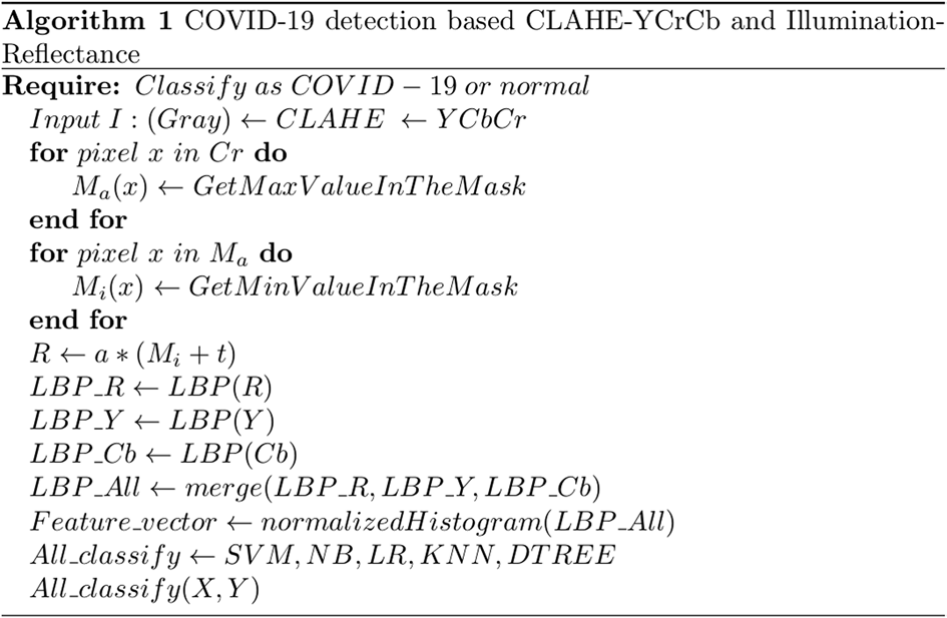


*R* is the estimated reflectance, *X*: training data, *Y*: class labels of *X*, *t* = 0.05 is a tiny positive value used to eliminate division zeros, and *a*= 1.1 is a small constant somewhat greater than 1 used to avoid an overly brilliant image [37].

### Local binary pattern (LBP)

LBP is a valuable method for extracting and categorizing textual information [9]. Maheshwari et al. [29] highlighted that LBP encodes the information about local pixel intensities in a binary-coded decimal value. Hence, LBP is an adequate texture descriptor. Image texture emphasizes color, intensity, and spatial arrangement information from an image or a designated location of interest.

$p_c$ as a pixel value in the central, *P* as the number of pixels in the close surroundings, and *r* as a neighborhood’s radius. Equation 9 shows the computational of LBP. LBPp,r: denotes the Local Binary Pattern value for a pixel with a radius of r and p sampling points (neighbors). For each sampling point (pi), S($p_i$-$p_c$) computes a binary value by comparing the intensity of the sampling point (pi) with the intensity of the central pixel (pc). If the intensity of pi is greater than or equal to pc, S($p_i-p_c$) is set to 1; otherwise, it is set to 0.

8 $$\begin{aligned} LBP_{p,r}&= \sum ^{p-1}_{i=1} S(p_i-p_c).2^i \ \end{aligned}$$

9 $$\begin{aligned} S(p_i-p_c)&= \Bigg \{ \begin{array}{cc} 1&: p_i \ge p_c \\ 0 &: p_i < p_c \end{array} \end{aligned}$$

**Fig. 3:**
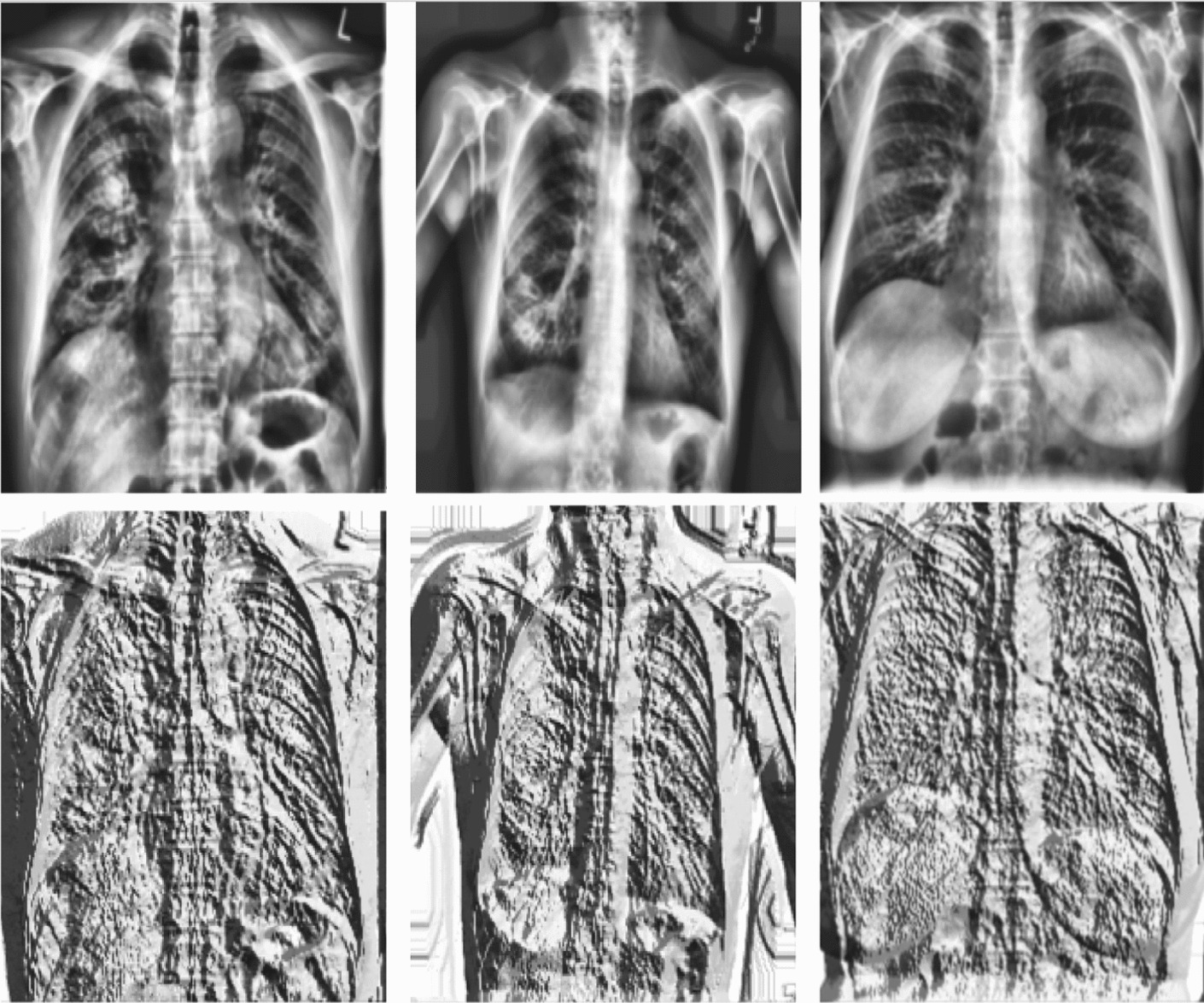
The top first row corresponds to COVID-19, normal, and pneumonia chest X-Ray images. The second row corresponds to the LBP image transformation of COVID-19, normal, and pneumonia

Figure 3 illustrates the local binary pattern (LBP) Transformation process applied to COVID-19, normal lungs, and pneumonia CXR images.

### COVID-19 chest X-ray detection

**Fig. 4:**
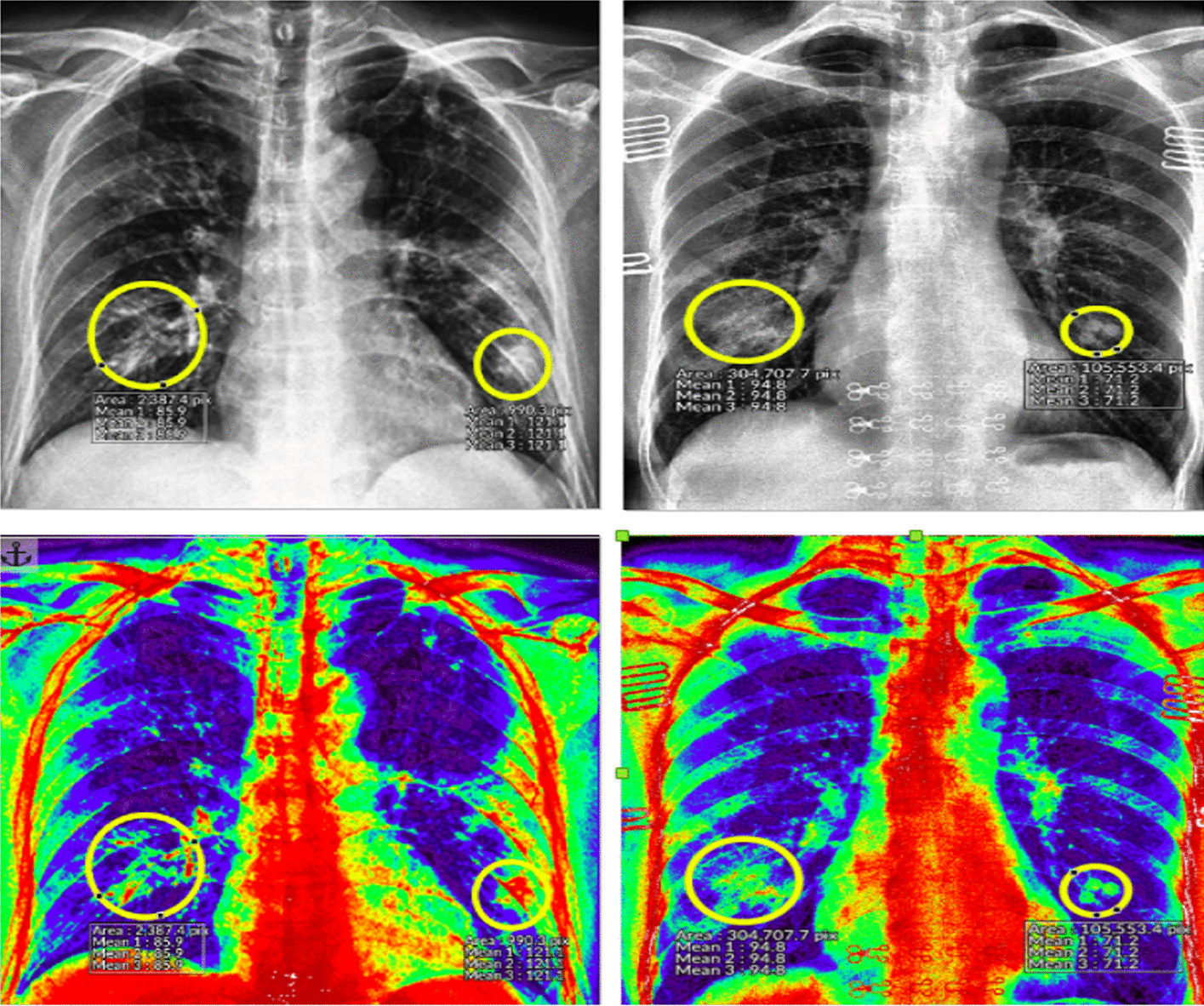
The first row depicts the CXR image with annotated abnormal regions (yellow circle). The second row highlights their corresponding rainbow transformations

Figure 4 depicts the abnormal regions of CXR (yellow circle). The first row highlights two different infected CXR images with COVID-19. At the same time, the second row depicts their corresponding rainbow transformations. A professional radiologist performed the annotations.

To detect COVID-19 in the input CXR images, we used CLAHE-YCrCb, LBP, and machine learning algorithms. Running multiple classifiers and comparing their performances has been a common strategy. We have considered the classifier with the best results as the best performer. Mubarak et al. [9] provide instant access to a variety of classification techniques, such as KNN, SVM, LDA [39], LR [40], DT [41], and NB [42].

## Experimental results

### Datasets description

We used three different CXR datasets from [16, 43, 44]. The first dataset [16] has 139, 190, and 200 images of COVID-19, pneumonia, and normal lungs, respectively. The second dataset [43] is constructed based on the below Table 1.

**Fig. 5:**
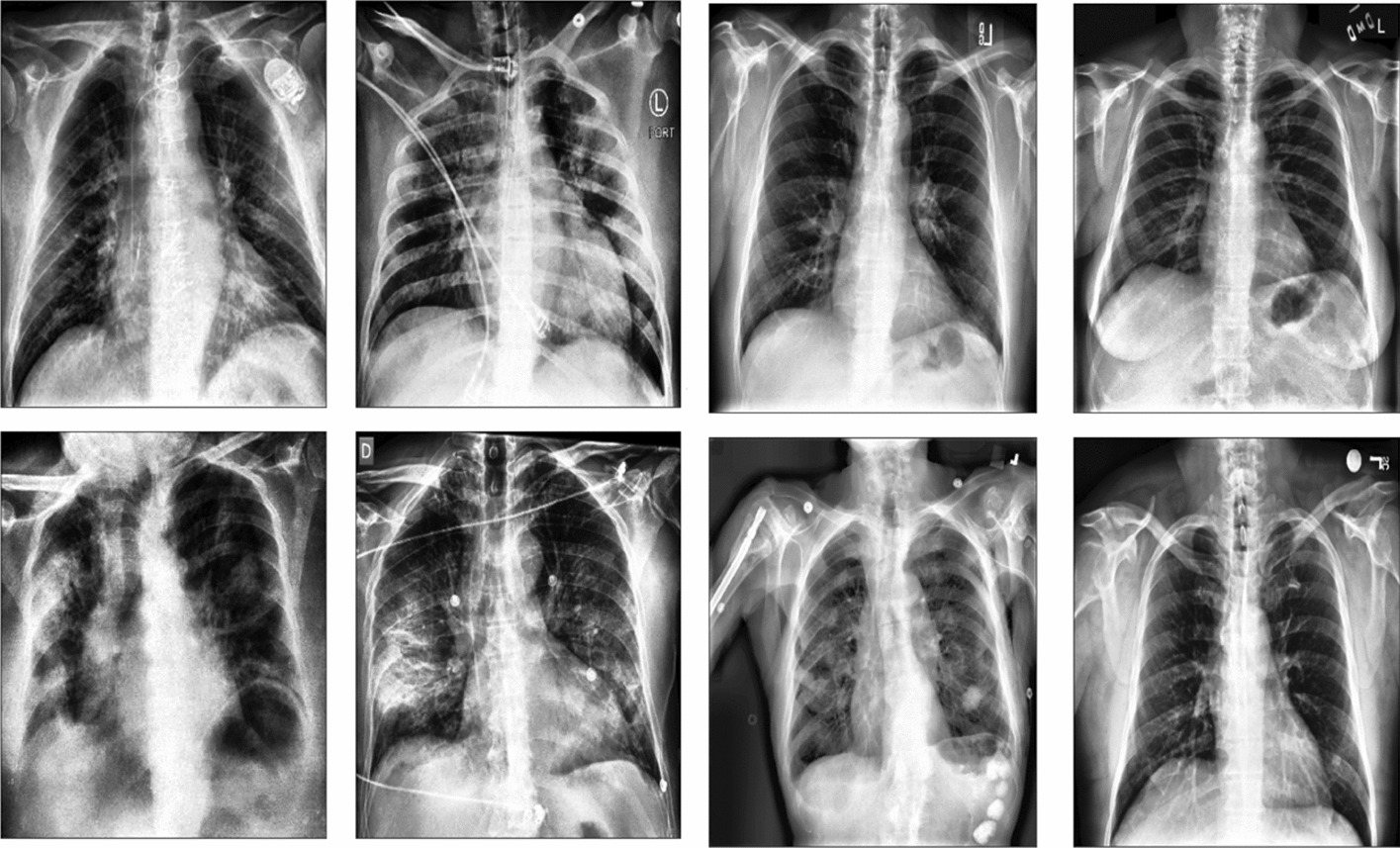
The first row depicts COVID-19 images. Whereas the second row shows normal images. These images were randomly selected from [16] dataset

**Fig. 6:**
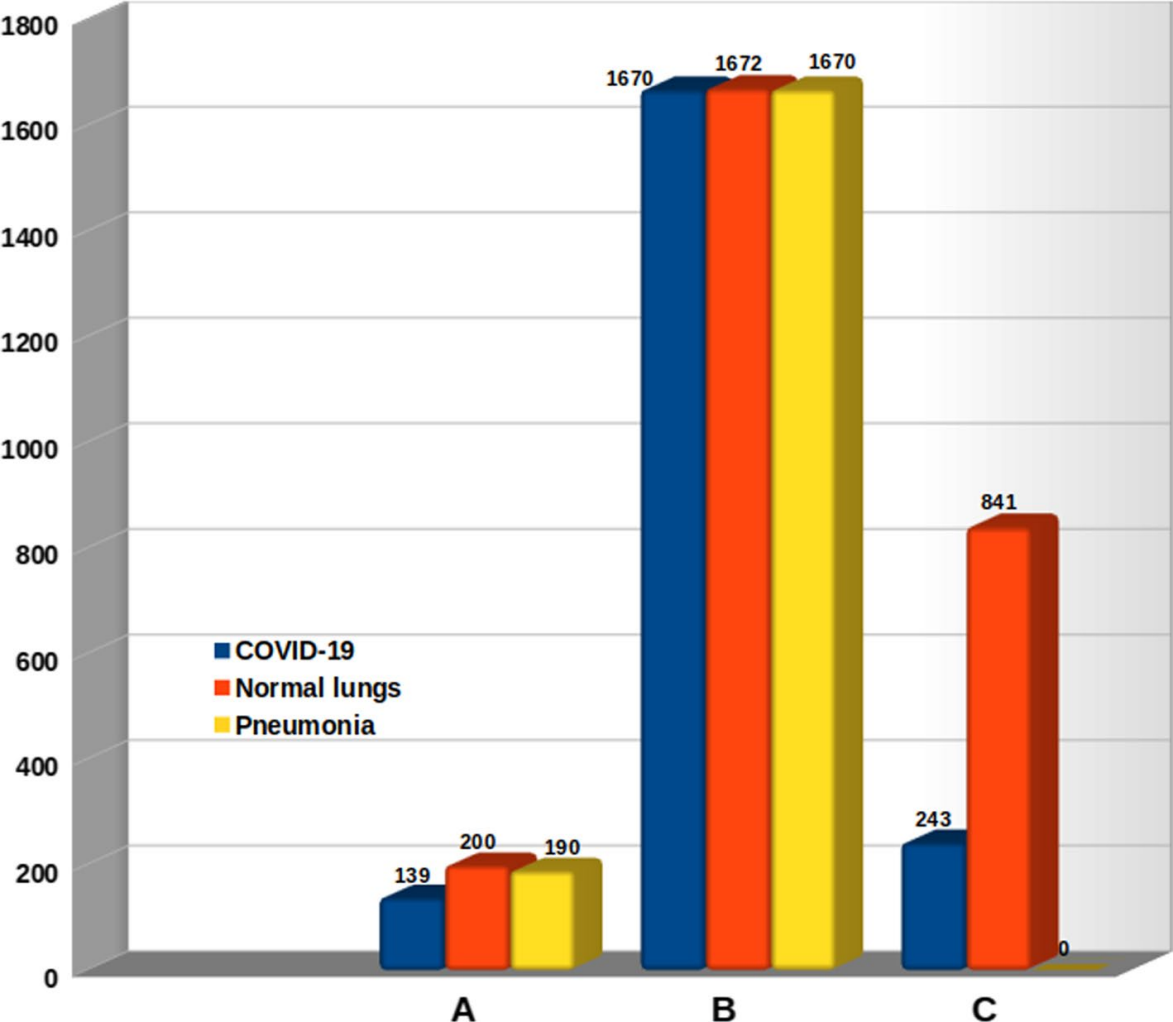
Data distribution (dataset A [16], dataset B [43], and dataset C [44])

Figure 5 highlights, in brief, a snapshot of used [16] datasets, Whereas Fig. 6 depicts the used datasets and their corresponding chest X-ray images.

**Table 1:** Description of used datasets by [43]

Datasets	Available images	Selected images
Ozturk et al.2020 [45]	COVID-19:125	COVID-19:125
	Normal:500	Normal:329
	Pneumonia:500	Pneumonia:325
Mooney,2020 [46]	Normal:1592	Normal:1343
	Pneumonia:4273	Pneumonia:1345
Chowdhury et al. [47], Rahman et al. [49]	COVID-19:3616	COVID-19:1545
	Normal:10,192	
	Total images:	COVID-19:1670
		Normal:1672
		Pneumonia:1670

The third dataset [44] has 841 negative and 243 positives (COVID-19) images.

### Performance metrics

The following metrics are used to evaluate the performance of the proposed model [48]: $t_p$ are COVID-19 CXR images correctly identified (True positives). Mistakenly identified images as COVID-19 are $f_p$ (False positives). Undetected COVID-19 chest X-Ray images classified as normal are $f_n$ (False negatives). CXR images appropriately recognized as normal are $t_n$ (True negatives). The true positive rate is denoted as $tp_r$, while the false positive rate is given by $fp_r$. Precision ($p_r$) specifies that a detected COVID-19 chest X-Ray image is genuinely a COVID-19 chest X-Ray image. In contrast, recall ($r_c$) denotes the probability of a valid COVID-19 CXR positive image being recognized. The $f_1$ score is a metric that combines $p_r$ and $r_c$ into a specific numerical. *acc* denotes the accuracy.

10 $$\begin{aligned} p_r&= \frac{tp}{t_p+f_p}, \;\;\;r_c = \frac{t_p}{t_p + f_n},\;\;\;\;f_1 =2\frac{p_r r_c}{p_r+ r_c} \end{aligned}$$

11 $$\begin{aligned} tp_r&= \frac{\#\;t_p}{\#\; COVID \ Chest\ X-Ray \;\; images} \end{aligned}$$

12 $$\begin{aligned} fp_r&= \frac{\#f_p}{\#\;non-COVID \ Chest\ X-Ray \;\;images} \end{aligned}$$

13 $$\begin{aligned} acc&= \frac{tp+tn}{t_p+t_n+f_p+f_n} \end{aligned}$$

### Analysis of running time and platforms

The platforms adopted include a Dell laptop with an Intel (R) Core (TM) i7-3540 M CPU @ 3.00GHz x 4. 64-bit with 8GB RAM. Python 3.7.6, Scikit-learn 0.23.1, and Ubuntu 18.04.3 LTS OS. Table 2 reports the running time in minutes. Training size = $70\%$ and test size = $30\%$

**Table 2:** Analysis of feature extraction, training time, prediction time, and the feature vector size

Datasets	Extracted features (M)	Training time (M)	Prediction (S)	Feature vector size
N-CLAHE-MEDICAL-IMAGES [16]	16.32	6.32	0.2	25
CoronaHack-chest X-ray-dataset [43]	47.39	12.54	0.8	25
DLAI3 Hackathon [44]	28.45	6.49	0.4	25

(M and S) depict minutes and seconds, respectively

## Results

The experiments are divided into three major sections: the first section used the dataset [16] to classify between Covid-19 and normal CXR images, and COVID-19 and pneumonia. The second section of the experiments focused on dataset [43] to classify between COVID-19 and normal CXR images. Finally, the dataset [44] was used in the last experimental section to detect COVID-19 and normal CXR images.

### Experiment (section one) using [16] dataset

**Fig. 7:**
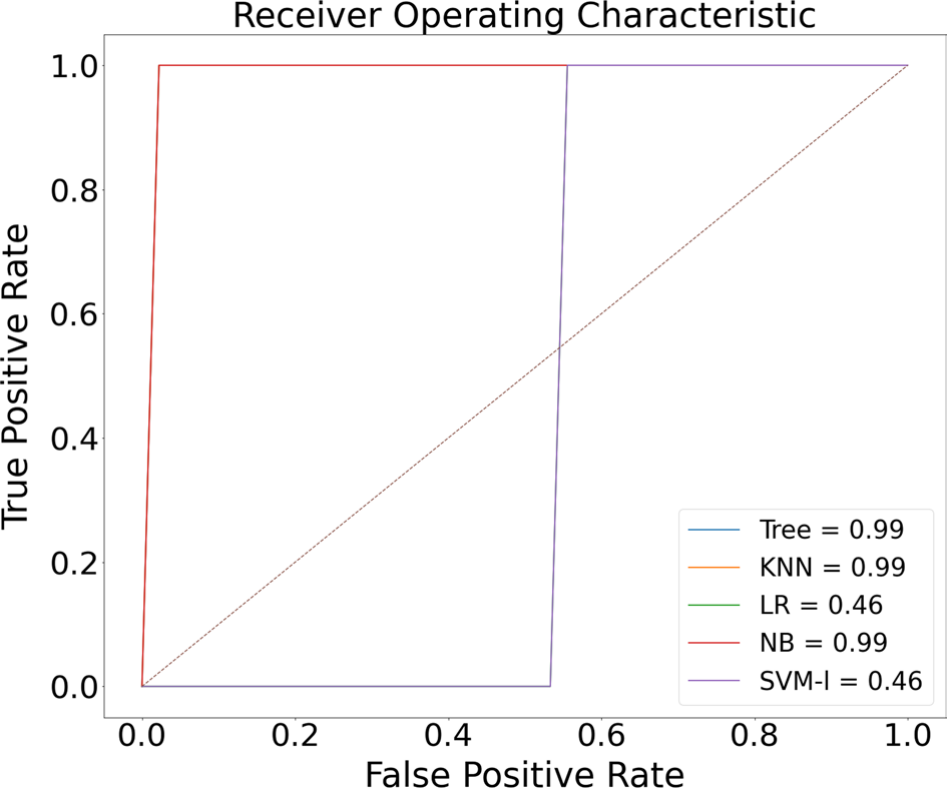
Receiver operating characteristic (ROC) curve

**Fig. 8:**
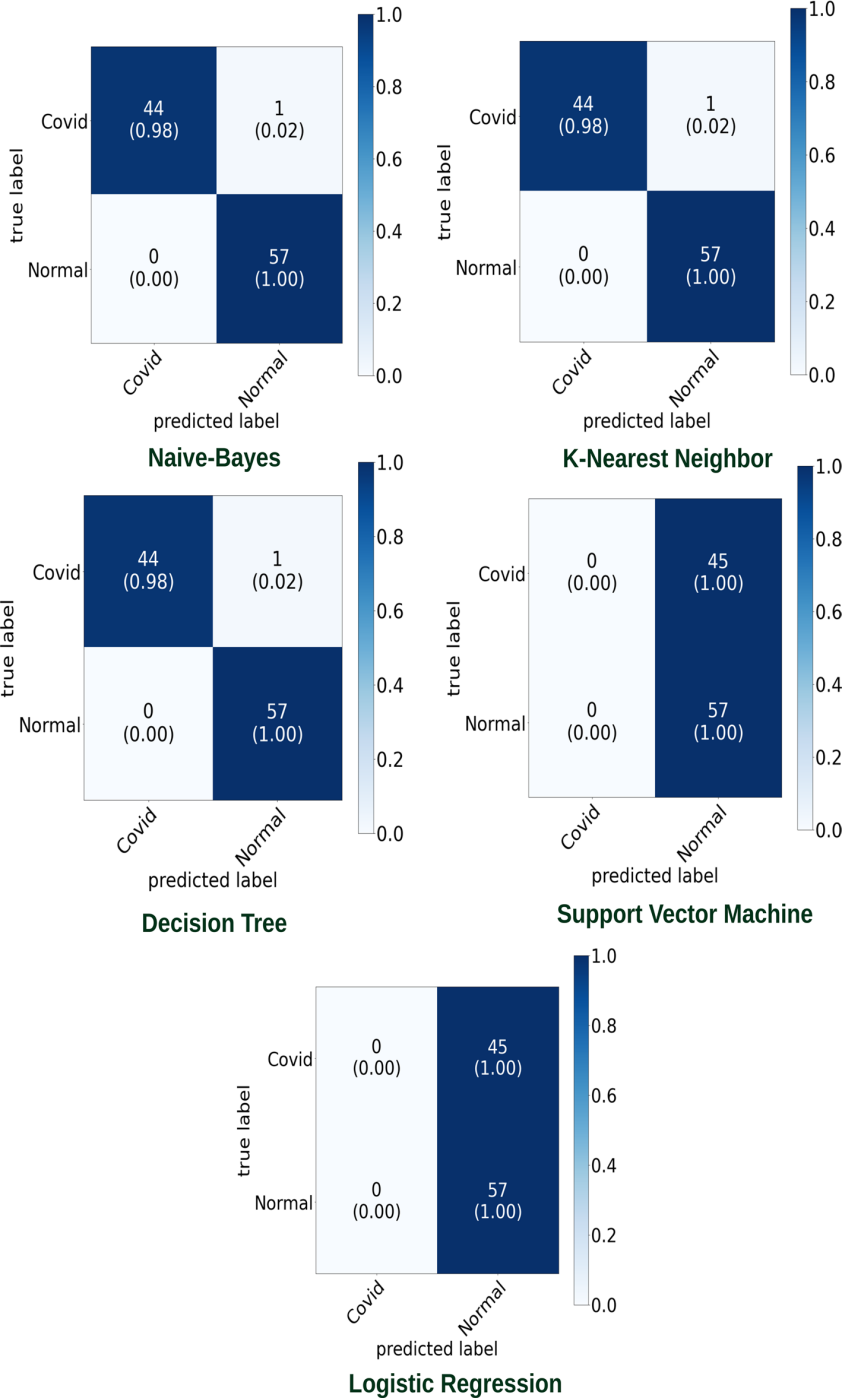
Confusion matrix portraying the plotted ROC in Fig. 7 using [16] dataset

The proposed model was initially trained using dataset [16]. We classified normal lungs and COVID-19 CXR images. Figures 7 and 8 highlight the graphical plots of the receiver operating characteristics (ROC) curve and the confusion matrix.

Table 3 portrays the obtained accuracy of different classifiers. DT, KNN, and Naive Bayes reported the highest accuracy of 99.01% each. LR and SVM reported a lower accuracy of 55.88% each.

**Table 3:** The accuracy report of Covid-19 and normal lung detection using [16] dataset

Classifiers	Accuracy (%)	Precision	Recall	F1-score
Logistic regression (LR)	55.88	0	–	–
Decision tree (DT)	99.01	97.77	100	98.87
K-nearest neighbor (KNN)	99.01	97.77	100	98.87
Naive Bayes (NB)	99.01	97.77	100	98.87
Support vector machines (SVM)	55.88	0	–	–

NB, KNN, and DT reported the highest accuracy of 99.01% each

We conducted another experiment, considering the same dataset [16]. This time, we were classifying COVID-19 and pneumonia CXR images.

**Fig. 9:**
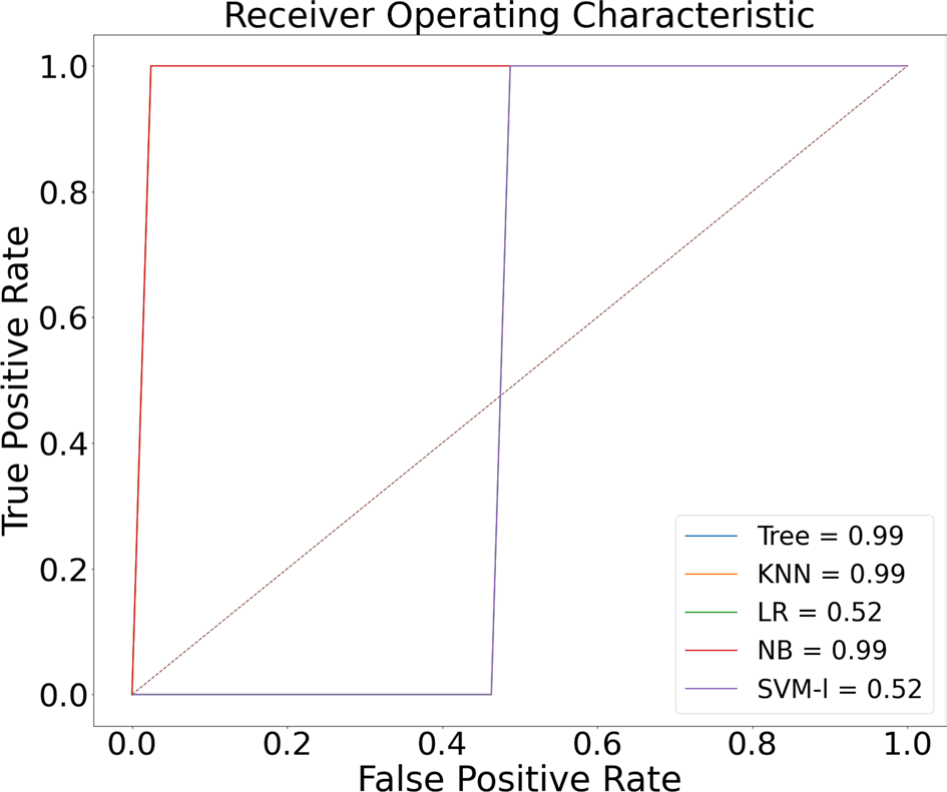
Graphical plot of ROC curve

**Fig. 10:**
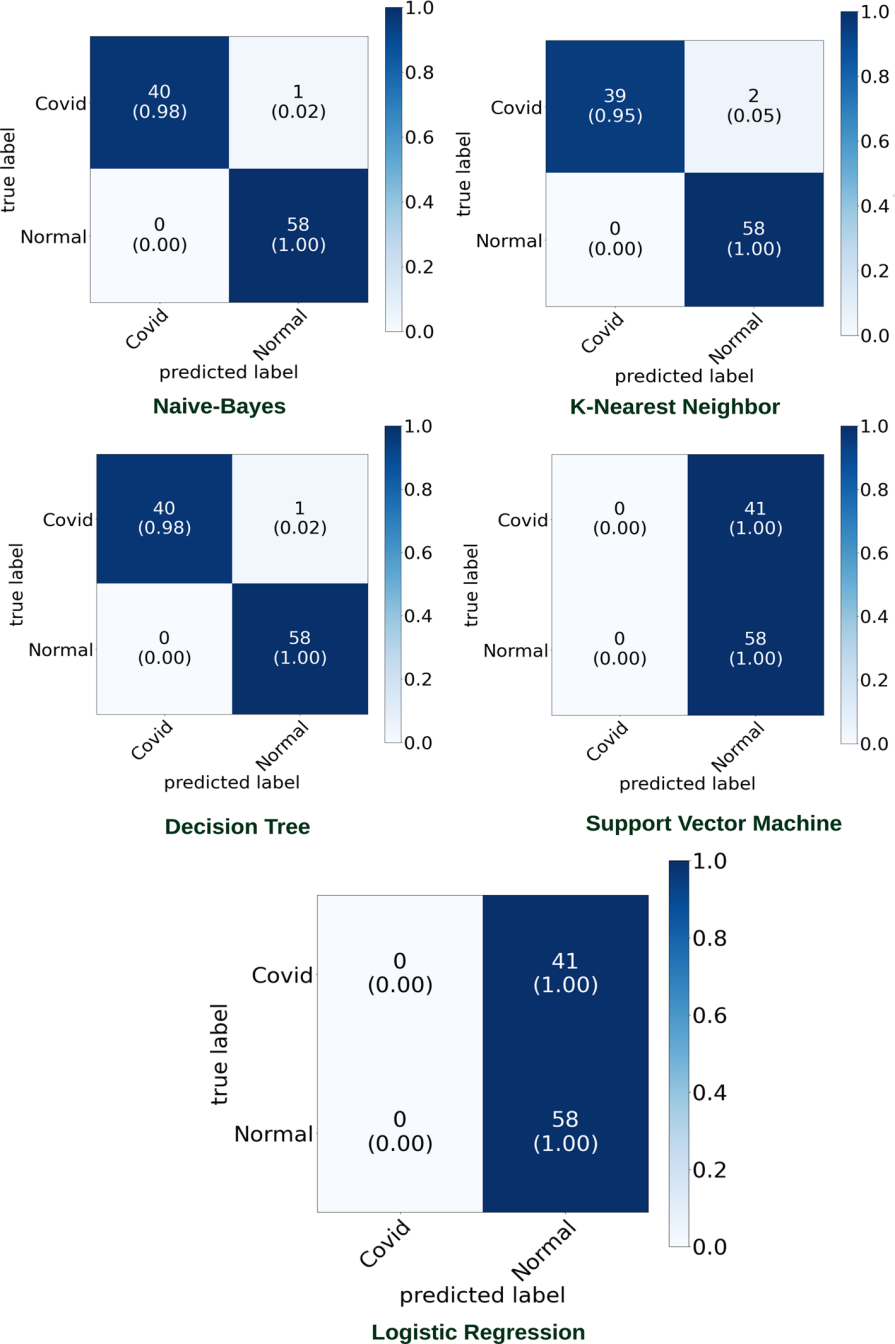
Confusion matrix portraying the plotted ROC in Fig. 9 using [16] dataset

Figures 9 and 10 highlight the graphical plots of the ROC curve and the confusion matrix.

Table 4 highlights the attained accuracy. Both the DT and NB reported an accuracy of 98.9% each. KNN reported an accuracy of 97.9%. LR and SVM reported a lower accuracy of 58.50% each.

**Table 4:** The accuracy report of Covid-19 and pneumonia diseases classification using [16] dataset

Classifiers	Accuracy (%)	Precision	Recall	F1-score
Logistic regression (LR)	58.50	0	0	–
Decision tree (DT)	98.9	97.56	100	97.56
k Nearest neighbor (KNN)	97.9	95.12	100	97.4
Naive Bayes (NB)	98.9	97.56	100	97.56
Support vector machines (SVM)	58.5	0	0	–

KNN reported an accuracy of 97.9%. NB and DT reported 98.9% accuracy each

### Experiment (section two) using [43] dataset

**Fig. 11:**
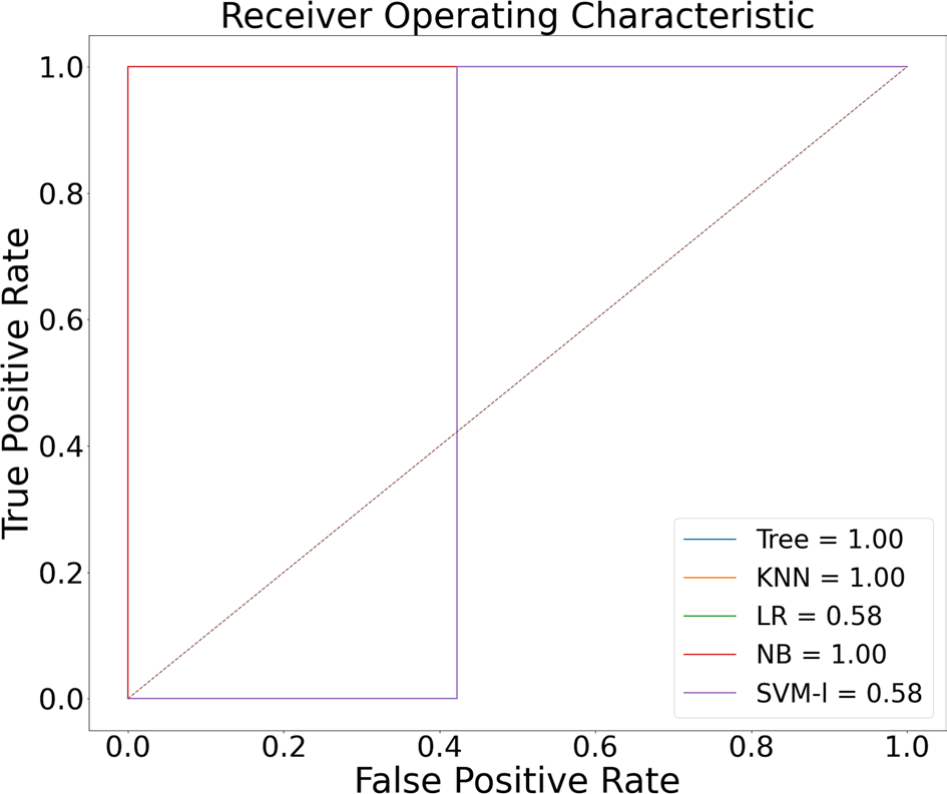
Receiver operating characteristic (ROC) curve

**Fig. 12:**
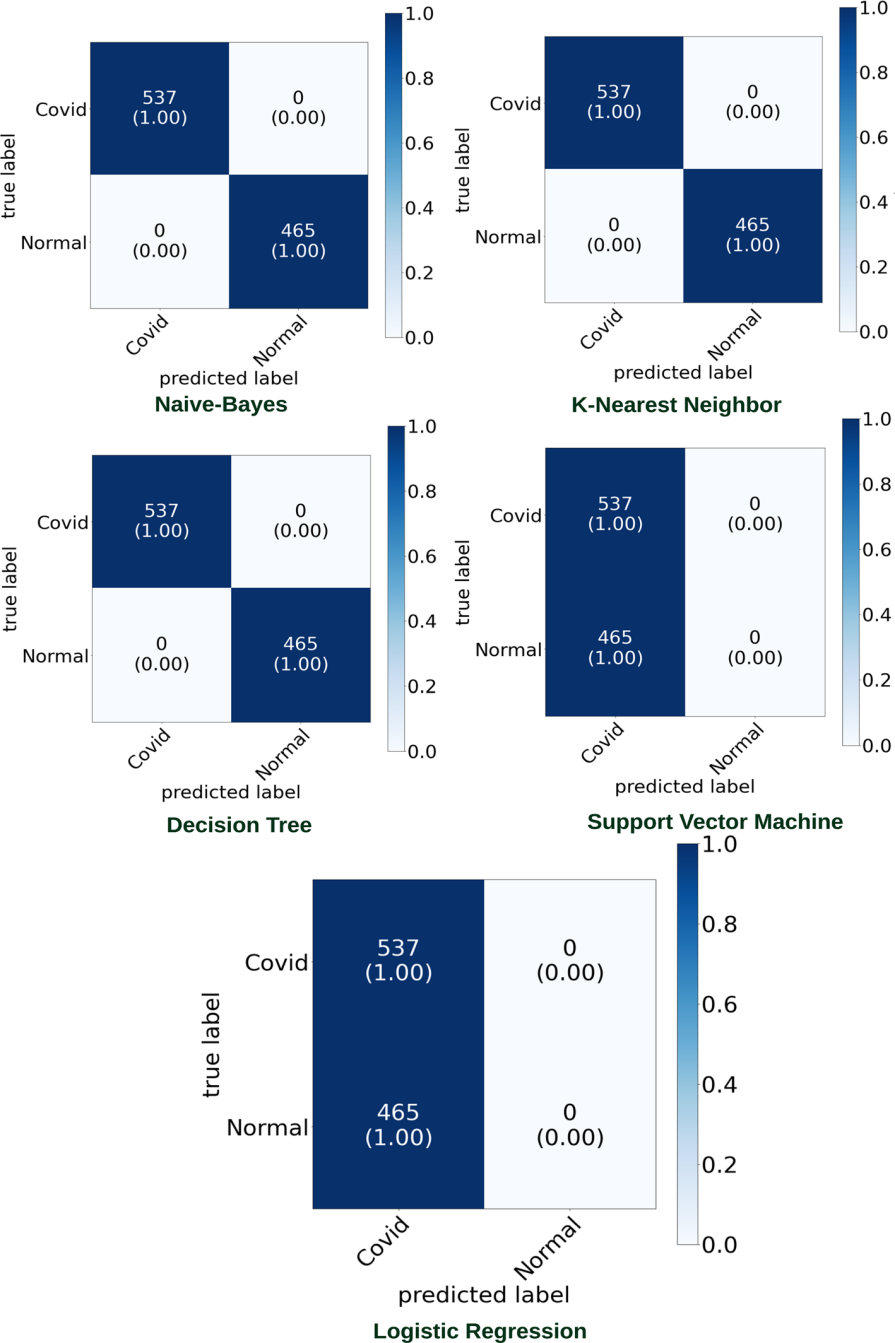
Confusion matrix portraying the plotted ROC in Fig. 11 using [43] dataset

The second part of our experiment considered [43] dataset to classify between Normal and COVID-19 CXR images. Figures 11 and 12 highlight the graphical plots of the ROC curve and the confusion matrix.

Table 5 outlines the reported accuracy. DT, KNN, and NB reported an accuracy of 100% each. While LR and SVM reported a lower accuracy of 53.59% each.

**Table 5:** The accuracy report of Covid-19 and normal lung detection using [43] dataset

Classifiers	Accuracy (%)	Precision	Recall	F1-score
Logistic regression (LR)	53.59	100	53.59	69.78
Decision tree (DT)	100	100	100	100
k-nearest neighbor (KNN)	100	100	100	100
Naive Bayes (NB)	100	100	100	100
Support vector machines (SVM)	53.59	100	53.59	69.78

Both DT, KNN, and NB reported an accuracy of 100% each

### Experiment (section three) using [44] dataset

**Fig. 13:**
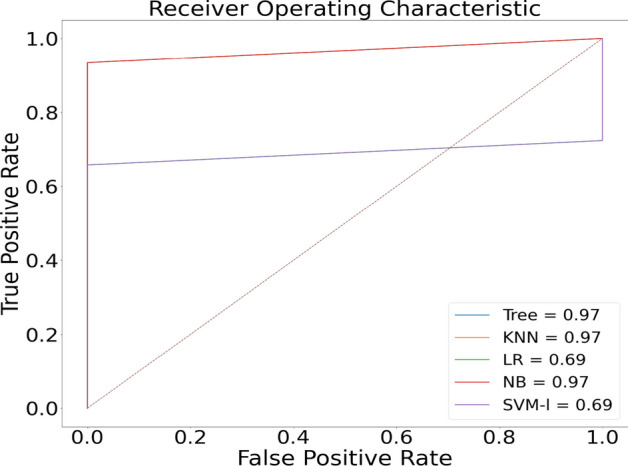
The receiver operating characteristic (ROC) curve

**Fig. 14:**
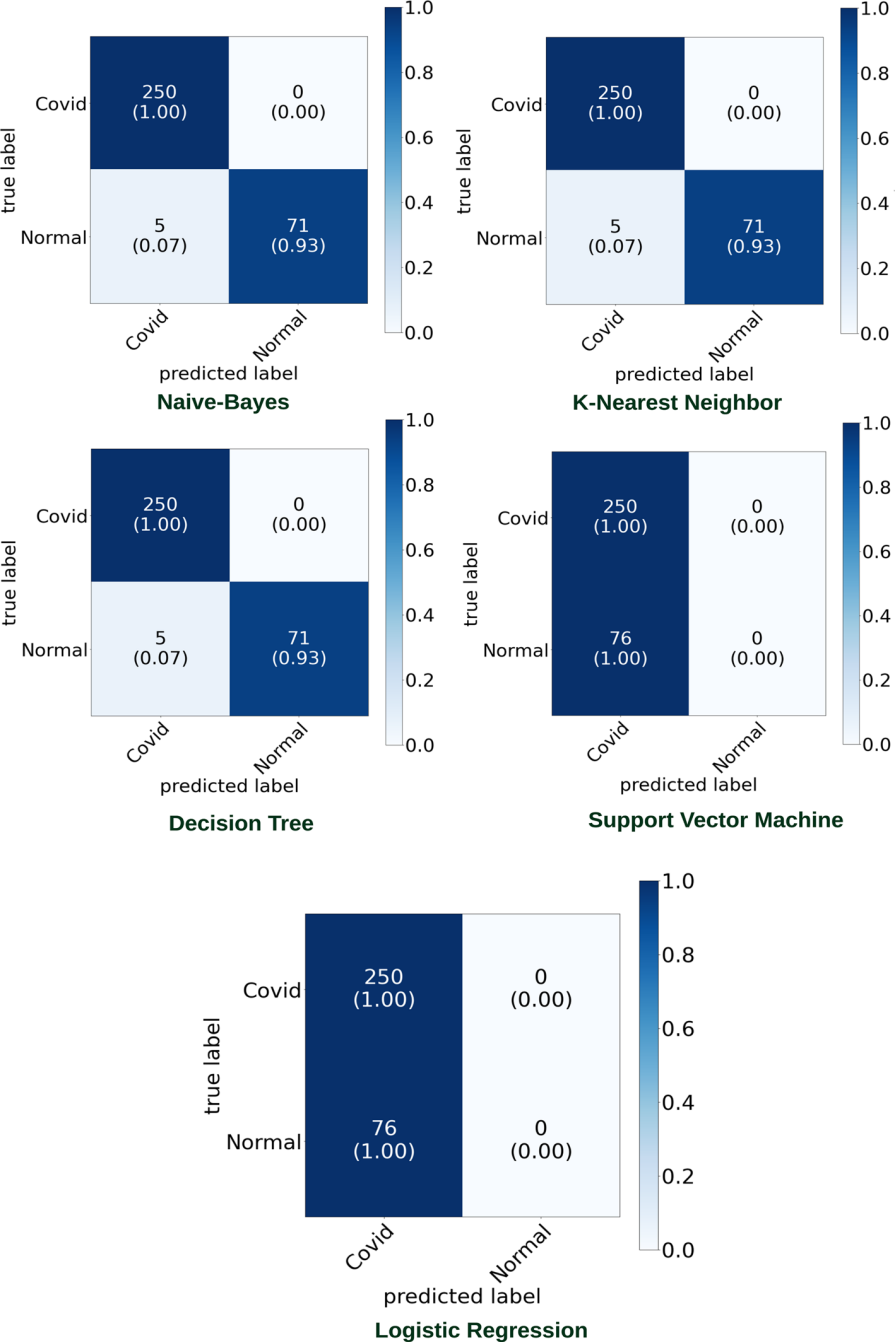
Confusion matrix portraying the plotted ROC in Fig. 13 using [44] dataset

The third part of the experiments considered [44] dataset to classify between normal and COVID-19 CXR images. Table 6 and Fig. 13 highlight both the model’s accuracy summary and the graphical plot of the ROC curve, respectively. While Fig. 14 outlines the confusion matrix.

**Table 6:** The accuracy report of Covid-19 and normal CXR image detection using [44] dataset

Classifiers	Accuracy (%)	Precision	Recall	F1-score
Logistic regression (LR)	76.68	100	76.68	86.80
Decision tree (DT)	98.46	100	98.03	99
Naive Bayes (NB)	98.46	100	98.03	99
k Nearest nearbor (KNN)	98.46	100	98.03	99
Support vector machines (SVM)	76.68	100	76.68	86.80

NB, DT, and KNN reported an accuracy of 98.46% each. While LR and SVM reported an accuracy of 76.68% each

**Fig. 15:**
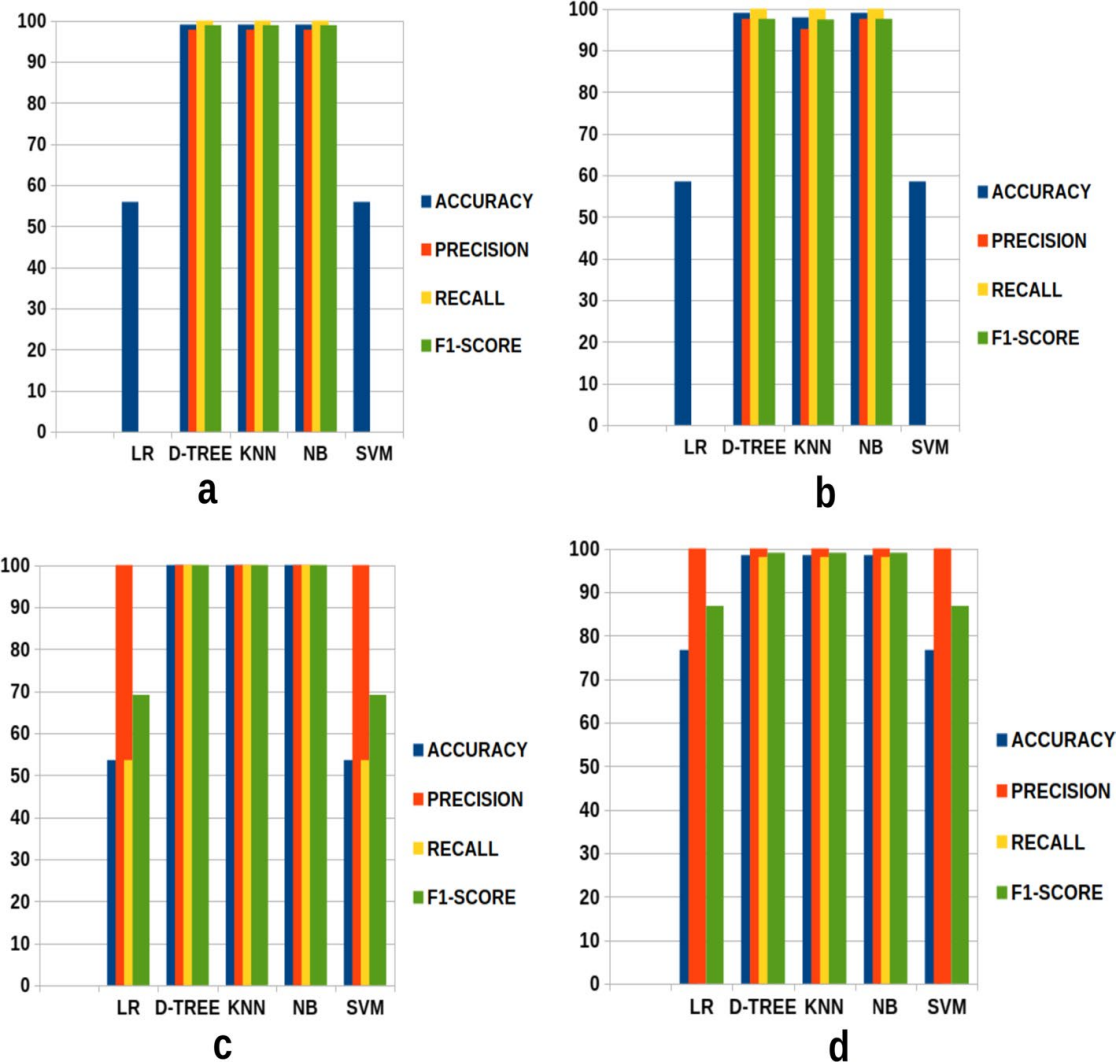
Graphs **a**, **b**, **c**, and **d** correspond to section one, section two, and section 3 experiments, respectively

Below Fig. 15 portrays all classifiers and their corresponding accuracy, precision, recall, and F1-Score, respectively. Graph (a) corresponds to the first part (section one) experiment to classify COVID-19 and normal CXR images. The second graph (b) depicts the classification results between COVID-19 and pneumonia CXR images. Graph (C) represents the experimental results of the second part (section two). Finally, graph (d) displays the results of the experiment’s third part (section three).

From Fig. 15, NB and DT outperformed other classifiers. While LR and SVM poorly detected COVID-19, normal, and pneumonia CXR images.

Both experiments, one and three, used imbalanced datasets. Therefore, to evaluate the measurement of the uncertainty of the proposed model, we used an accuracy confidence interval and PR curve.

**Fig. 16:**
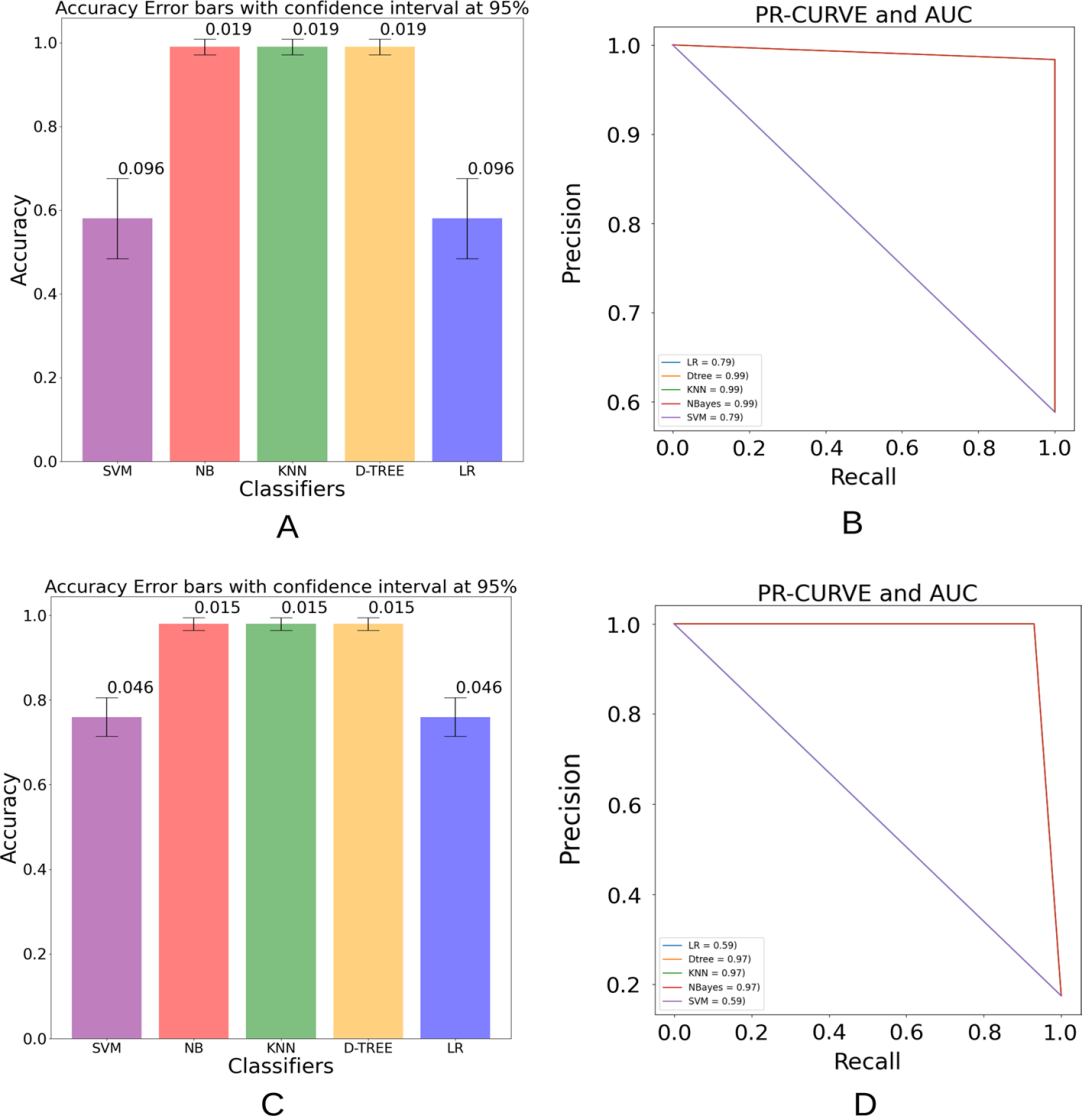
Graphs **A** and **B**, **C** and **D** correspond to the confidence interval and PR curve of the performed experiments, section one and three respectively

Figure 16, (A and B) highlights the Confidence interval and PR curve of the performed section one experiment classifying Covid-19 and normal images, respectively. Whereas (C and D) portray the confidence interval and PR curve of the performed section three experiment, classifying Covid-19 and normal, respectively.

## Comparative results

The proposed model is computationally efficient (see Table 7). It has a simple model architecture (see Table 8), and uses a small feature vector size (see Table 9).

Table 7 demonstrates the platform and obtained accuracy. Taking into consideration the work performed by [16], they adopted an Intel Xeon Gold 6150 2.7GHz 18-core (16 cores enabled) server with 24.75MB L3 Cache, (Max Turbo Freq. 3.7GHz, Min 3.4GHz). The GPU on this server was an NVIDIA Quadro P5000 (2,560 Cores, 16GB Memory). RAM on the server was Three hundred and sixty GB (Six channels).

Our proposed model uses a Dell laptop with an Intel (R) Core (TM) i7- 3540 M CPU @ 3.00GHz x 4. 64-bit with 8GB RAM. Python 3.7.6, Scikit-learn 0.23.1, and Ubuntu 18.04.3 LTS OS.

**Table 7:** Comparative results of our proposed model with Horry et al. [16], regarding accuracy and used platform

Used models	Accuracy (%)	Used platform
Horry et al. [16]	86	Intel Xeon Gold 6150 2.7GHz 18 cores (Server)
Our proposed model	100	Dell intel(R)core (Laptop)

Table 8 highlights the reported accuracy and the model descriptions. El-Sayed et al. [43] used the RESCOVIDTCNNet model. This integrates the empirical wavelet transform (EWT), temporal convolutional neural network (TCN), dilated Causal Convolution Layer, and residual block. They reported an accuracy of 100%. Rahman et al. [49] adopted a transfer learning model (ChexNET). They reported an accuracy of 96.29%. Chowdhury et al. [47] used the DensNet201 transfer learning model. An accuracy of 99.70% was computed.

In contrast, our proposed model uses a simple architecture of CLAHE, YCrCb, LBP, and machine learning algorithms to classify our CXR images.

**Table 8:** Comparative results between the proposed model with El-Sayed et al. [43] dataset

Used models	Accuracy (%)	Model descritpions
Rahman et al. [49]	96.29	ChexNet
El-Sayed et al. [43]	100	EWT+TCN Structure + Dilated Causal Convolution Layer + Residual Block
Chowdhury et al. [47]	99.70	DensNet201
Our proposed model	100	YCrCb+LBP + Machine Learning Algorithms

Taking into consideration the accuracy and the model descriptions

Table 9 depicts reported accuracy, trainable parameters, and the feature vector size. The work performed by [44] used estimated trainable parameters of 2112. These methods compute the trainable parameters [50, 51].

**Table 9:** Comparative results between the proposed model with Chenqi li et al. [44] datasets considering the accuracy, trainable parameters, and feature vector size

Used models	Accuracy (%)	Trainable parameters/feature vector size
Khalifa et al. [52]	97.4	–
Chenqi li et al. [44]	98	2112 Trainable-parameters
Our proposed model	98.46	**25** Feature vector-size

## Conclusion

This paper presents a novel method to detect COVID-19, Normal, and pneumonia using CXR images. The proposed method is based on Contrast Limited Adaptive Histogram Equalization, Illumination–Reflectance model, and LBP. This method takes input chest X-ray images and enhances them using the CLAHE algorithm. The output images from CLAHE are converted into YCrCb color space. The reflectance component is estimated using the Illumination–Reflectance model from Cr. Finally, the Local Binary Patterns (LBP) histogram generated from reflectance and YCb is used as the feature vector. Experimental results from three publicly available datasets reported accuracy of 99.01%, 100%, and 98.46%, respectively. Our model is computationally efficient, using a small feature vector size and less running time. Emerging nations can use this prototype where radiologists need more supply.

Our future work will explore other modalities, such as CT scans, ultrasounds, and chest MRIs. We will investigate multi-classification tasks between COVID-19, normal, and pneumonia, also exploring why SVM and LR are ineffective. Additionally, we will utilize image blob visualization techniques to precisely and accurately locate the infected area. Ultimately, we hope to develop an AI model that can be integrated with electronic health records (EHRs) to extract critical clinical data, including vital signs, lab results, and patient demographics, and combine it with chest X-ray images to enhance the accuracy of COVID-19 diagnosis and interpretability.

## Data Availability

The three datasets used during the current study are available in the following repositories: 1 N-CLAHE-MEDICAL-IMAGES, https://github.com/mhorry/N-CLAHE-MEDICAL-IMAGES/tree/master/CXR/C-P-N 2 CoronaHack -Chest X-Ray-Dataset, https://www.kaggle.com/datasets/praveengovi/coronahack-chest-xraydataset 3 DLAI3 Hackathon, https://www.kaggle.com/c/dlai3/ The coding materials used during the current study are available in the CLAHE-YCrCB-LBP-COVID-19-Detection repository. https://github.com/rukuprince/CLAHE-YCrCB-LBP-COVID-19-Detection.

## Abbreviations

C.X.R: Chest X-rays
LBP: Local binary pattern
HE: Histogram equalizer
CLAHE: Contrast limited adaptive histogram equalization
R.O.C: Receiver operating characteristics
CNN: Convolutional neural network
TL: Transfer learning
AUC: Area under the curve
PR: Precision–recall
CI: Confidence interval
